# A Strategy for Screening Monoclonal Antibodies for *Arabidopsis* Flowers

**DOI:** 10.3389/fpls.2017.00270

**Published:** 2017-02-28

**Authors:** Qian Shi, Lian Zhou, Yingxiang Wang, Hong Ma

**Affiliations:** ^1^State Key Laboratory of Genetic Engineering and Collaborative Innovation Center of Genetics and Development, Ministry of Education Key Laboratory of Biodiversity Science and Ecological Engineering and Institute of Biodiversity Sciences, Institute of Plants Biology, Center for Evolutionary Biology, School of Life Sciences, Fudan UniversityShanghai, China; ^2^Institutes of Biomedical Sciences, Fudan UniversityShanghai, China

**Keywords:** flower development, antibody library, *Arabidopsis*, monoclonal antibody, molecular marker

## Abstract

The flower is one of the most complex structures of angiosperms and is essential for sexual reproduction. Current studies using molecular genetic tools have made great advances in understanding flower development. Due to the lack of available antibodies, studies investigating the localization of proteins required for flower development have been restricted to use commercial antibodies against known antigens such as GFP, YFP, and FLAG. Thus, knowledge about cellular structures in the floral organs is limited due to the scarcity of antibodies that can label cellular components. To generate monoclonal antibodies that can facilitate molecular studies of the flower, we constructed a library of monoclonal antibodies against antigenic proteins from *Arabidopsis* inflorescences and identified 61 monoclonal antibodies. Twenty-four of these monoclonal antibodies displayed a unique band in a western blot assay in at least one of the examined tissues. Distinct cellular distribution patterns of epitopes were detected by these 24 antibodies by immunofluorescence microscopy in a flower section. Subsequently, a combination of immunoprecipitation and mass spectrometry analysis identified potential targets for three of these antibodies. These results provide evidence for the generation of an antibody library using the total plant proteins as antigens. Using this method, the present study identified 61 monoclonal antibodies and 24 of them were efficiently detecting epitopes in both western blot experiments and immunofluorescence microscopy. These antibodies can be applied as informative cellular markers to study the biological mechanisms underlying floral development in plants.

## Introduction

Köhler and Milstein (1975) generated the first monoclonal antibody. Since then, 100s and 1000s of monoclonal antibodies have been widely used in various fields of medical and biological research (Weiner, 2015; Teo et al., 2016). In the 1980s, Aris and Blobel built an antibody library using purified nuclei proteins from yeast (Aris and Blobel, 1988). This library screen directly contributed to the discovery of the nuclear pore complex, promoting further studies of its role in regulating intracellular signaling pathways (Aris and Blobel, 1988). Because monoclonal antibodies can bind to an epitope with high degrees of specificity and sensitivity, they have become more and more important for detecting protein subcellular localization in many organisms, including plants.

The flower is the reproductive structure of angiosperms, producing the male and female gametes and providing the physical and nutritional environment for seed formation (Soltis et al., 2004). The basic structure of most flowers consists of four whorls from the outer to the inner whorl: sepals, petals, stamens and carpels (Smyth et al., 1990; Ma, 2005). In recent decades, molecular genetic studies have made great progress in understanding regulatory mechanisms of flower development, such as the classic ABC model (Alvarez-Buylla et al., 2010; Bowman et al., 2012). Due to the lack of available antibodies against the proteins underlying ABC model, information about their subcellular localization remains limited. At the same time, knowledge regarding subcellular structures in floral tissues is very limited due to the lack of cellular markers, especially for structures that are not conserved from animals and fungi, which have been studied using cell biological tools much more extensively.

To generate monoclonal antibodies that can be used as molecular markers for studying cellular structures during flower development, we constructed a library of monoclonal antibodies using total proteins from the inflorescences of *Arabidopsis thaliana*. Our initial screens using western blot (WB) identified a total of 61 antibodies that displayed bands in *Arabidopsis* total proteins. 24 of these antibodies detected a single weight protein band of various sizes from floral protein extracts. We then performed WB using total proteins extracted from different organs such as stems, leaves and inflorescences and grouped these antibodies into three categories: tissue-specific, preferential, and broad expression. Further characterization of these antibodies by performing immunofluorescence microscopy in *Arabidopsis* inflorescence paraffin sections revealed that different protein signals specifically localized in *Arabidopsis* inflorescence, with some exhibiting expression in specific cell layers. Finally, we used immunoprecipitation (IP) to enrich putative antigens (or antigen complexes) and performed mass spectrometry (MS) analysis to discover the target antigens of these antibodies. Taken together, this is the first time that monoclonal antibodies were generated using total plant proteins as antigens. Furthermore, the identified antibodies could be used as molecular markers for studying floral organ development.

## Materials and Methods

### Plant Material and Flower Protein Extraction

The *A. thaliana* wild-type plant used in this study was the Col ecotype. The plants were grown in the greenhouse with 16 h of light and 8 h of darkness under constant 22°C. The flower from stages 1–12 were collected and ground to a fine powder in liquid nitrogen; the proteins were extracted by using the extraction buffer [100 mM Tris-HCl, pH = 7.5; 300 mM NaCl; 2 mM EDTA, 10% Glycerol; 0.1% Triton X-100; 1x complete protease inhibitor (11697498001, Roche, USA)]. The protein–buffer mixture was centrifuged at 13000 rpm for 10 min at 4°C. The supernatant was collected. The protein concentration of the supernatant was measured by using a Bio-Rad Protein Assay Kit (Bio-Rad, Berkeley, CA, USA). This extract was then used to immunize mice.

### Generation of the Monoclonal Antibody Library toward Proteins from Flower

Total proteins were exacted as above and diluted to a concentration of 1 mg/mL to be used as the antigen. The antigen was emulsified with Complete Freund’s adjuvant (CFA) with a volume ratio of 1:1 before immunizing the mice. Monoclonal antibodies were generated using standard method as previously described (Yokoyama et al., 2013; Greenfield, 2014). Briefly, BALB/c mice were immunized with 150 ng of antigen, followed by a booster of 150 ng on day 14 intervals and then injected on day 28. The mouse’s spleen cells (1.0 × 10^7^/mL) were isolated and fused with mouse P3X63Ag8.653 cell line (2.0 × 10^7^/mL) to generate the hybridoma cells. Polyethylene glycol (PEG) was used as adjuvants in later immunization steps. The hybridoma cells were screened by western blot twice. Positive cells were picked for sub-cloning by limiting dilution. The hybridoma cell clones were also screened by western blot twice. Positive clones were then collected for expansion culture. The supernatant of the antibody was harvested and purified using protein A.

### Immunoblotting and Immunoprecipitation

The total protein used was the same as described above. For immunoblotting, the proteins were separated on a 4–15% polyacrylamide gradient gel (4561086, Bio-Rad, USA) and transferred onto a nitrocellulose membrane (10600002, Amersham, USA). The membrane was blocked with 5% non-fat milk (9999, Cell Signaling, USA) in TBST and incubated with the monoclonal antibodies (1:500 dilution) over night at 4°C. The membrane was washed three times for 5 min each with TBST. HRP-conjugated anti-mouse IgG secondary antibody was added for 1 h at room temperature. The membrane was washed three times again with TBST before being treated with ECL (RPN3243, GE Healthcare, USA) and scanned by a Typhoon scanner (FLA 9500, GE Healthcare, USA). For immunoprecipitation, the antibodies were added to the protein extract at the previously described concentration and incubated for 2 h at 4°C before incubation with protein A-conjugated beads for another 1 h. The beads were collected by centrifugation at 2000 *g* for 2 min at 4°C and washed three times with TBST before boiling in SDS loading buffer for 10 min. The samples were then analyzed by 4–15% SDS-PAGE and silver staining as described (Chevallet et al., 2006).

### Immunofluorescence Microscopy

Immunofluorescence staining was performed as described previously (Wang et al., 2012). The slides were blocked with goat serum (AR0009, Boster Biological Technology, China) at 37°C for 30 min, followed by the incubation with one of the monoclonal antibodies (1:500 dilution) at 4°C overnight. The slides were then washed three times with PBS for 10 min each before incubating with goat anti-Mouse IgG (H+L) Secondary Antibody, Alexa Fluor^®^ 488 conjugate (A-11001, Invitrogen, USA), at a 1:1000 dilution in PBS for 1 h at room temperature. After washing three times with PBS, the slides were stained with 1.5 mg/mL 4,6-diamidino-2-phenylindole (DAPI) in vectashield antifade medium (H-1200, Vector Laboratories, USA). The slides were imaged using an AxioCam HRc (Zeiss) camera.

### Identification of the Antigens by Mass Spectrometry

After the silver staining (Chevallet et al., 2006), the targeted band was excised for gel digestion by trypsin as described previously (Shevchenko et al., 2006). After digestion, the extracted peptides were analyzed by a Finniqan LTQ mass spectrometer (Thermo, USA) coupled with a surveyor HPLC system (Thermo, USA).

## Results

### Generation of a Monoclonal Antibody Library Using Total Proteins from *Arabidopsis* Inflorescences as Antigens

**FIGURE 1:**
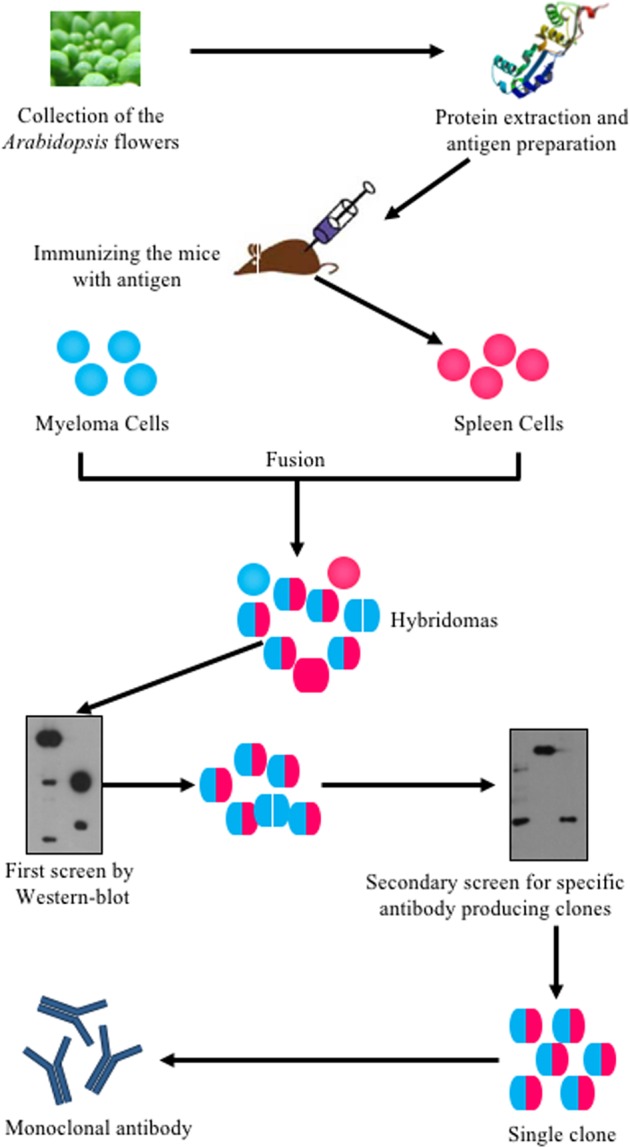
**Flowchart of systematically generating monoclonal antibodies using *Arabidopsis* flower total proteins as antigen.** *Arabidopsis* flowers at stages 1–12 were collected. The total protein was extracted and quantified using a Bio-Rad Protein Assay Kit. The proteins were then prepared for mice immunization. The spleen cells from immunized mice were fused with myeloma cells to produce hybridoma cells. The hybridoma cells were screened twice by western blot. Positive clones were kept for antibody production.

*Arabidopsis* is a widely used model system for plant molecular genetics and its flower development has been extensively studied in the last three decades (Chang and Meyerowitz, 1986; Alvarez-Buylla et al., 2010; Irish, 2010; Bowman et al., 2012). However, only a few antibodies were successfully produced to trace floral proteins and to study floral development. To understand molecular mechanisms underlying flower development, previous studies used transgenic plants expressing a fusion of the target protein with an epitope tag to examine the target protein level, modification or localization with commercial tag antibodies (Terpe, 2003). However, this approach is inefficient for detecting the same protein in different genetic backgrounds, especially due to the time and effort needed to introduce the transgene into various backgrounds. Therefore, as many proteins are discovered with crucial roles in development, specific antibodies have become more and more important. In this study, we used a procedure (**Figure 1**) to generate a monoclonal antibody library against the total proteins extracted from *Arabidopsis* stage 1–12 inflorescences, as defined previously (Smyth et al., 1990; Alvarez-Buylla et al., 2010). Total proteins were used to immunize the mice and the spleen cells from each immunized mouse was isolated and fused with myeloma cells to generate hybridoma cells, which were then cultured in HAT (hypoxanthine-aminopterin-thymidine) medium. The culture media of these hybridoma were tested twice by WB using total floral proteins to determine whether the hybridoma produced antibodies that recognized floral proteins. A single clone was then selected from each antibody-positive culture and the culture media of the single clones were tested again by WB to confirm the production of antibodies recognizing flower proteins. All positive clones were then selected for subsequent culturing. Thus, we generated about 1000 individual clones from which 61 of those clones specifically recognized floral proteins in a WB assay. Protein A was then used to purify these antibodies for further study.

### Testing Monoclonal Antibodies for Organ Specificity by Western Blot

**FIGURE 2:**
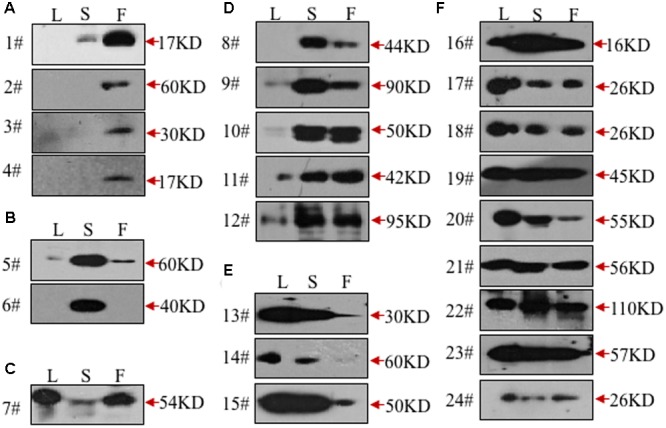
**Validation of the monoclonal antibodies by western blot.** All the monoclonal antibodies were validated by western blot using total protein extracted from leaves (L), stems (S), or flowers (F). According to the tissue specificity, the antibodies were divided into six groups. The antibodies in the first two groups recognized proteins mainly in flowers **(A)** or stems **(B)**. In the next three groups, the targeted proteins were mainly from leaves and flowers **(C)**, stems and flowers **(D)**, leaves and stems **(E)**, respectively. All three tissues were detected with signals in the last group **(F)**.

To validate the ability of the 61 monoclonal antibodies to detect floral proteins and examine their specificity for particular proteins, we performed WB assays using total proteins extracted from *Arabidopsis* leaves, stems and inflorescences. According to the protein specificities recognized by the individual antibodies (**Figure 2**, Supplementary Figure S1, and **Table 1**), 24 of the 61 antibodies were able to detect a single weight protein band and were selected for subsequent analyses. According to similarity or difference of signal profiles, the 24 antibodies were divided into six groups from A to F (for convenience, those antibodies were named as No. 1 to No. 24, hereafter). Group A contains four antibodies, No. 1–4, recognized an organ-specific (flower) protein. Group B contains two members: No. 5 and No. 6, which detected a protein at higher levels in stems. The antigen for No.6 was highly expressed in stems compared to leaves and flowers, whereas No. 7 showed an opposite pattern with the recognition of a protein expressed at higher levels in leaves and flowers than in stems (**Figure 2C**). Group D includes five antibodies (No. 8–12), for which all antigens had relatively high expression levels in both stem and flower, but with low or undetectable levels in leaves (**Figure 2D**). In Group E, three antibodies (No. 13–15) detected proteins at higher levels in the leaves and stems than in the flowers (**Figure 2E**). Finally, Group F includes nine antibodies, whose antigens were similarly expressed in all the three examined organs (**Figure 2F**). We also estimated the signal intensity of WB bands for each antibody by Image J, as shown from 1A to 5A (**Table 1**). Together, the WB data demonstrated that the 24 antibodies reported here have antigens that are either specific to certain organs or are ubiquitously expressed.

**Table 1:** A summary of the 24 monoclonal antibodies.

Type	Clone NO.	Signal specificity			MW (kDa)	Signal intensity
		Leave (L)	Stem (S)	Flower (F)		
I	1			√	17	AAA
	2			√	60	A
	3			√	30	A
	4			√	17	A
II	5		√		58	AAA
	6		√		40	AAA
III	7	√		√	54	AAAA
IV	8		√	√	44	AA
	9		√	√	90	AAA
	10		√	√	50	AAAA
	11		√	√	42	AAA
	12		√	√	95	AAAA
V	13	√	√		30	AAAAA
	14	√	√		60	A
	15	√	√		50	AAAAA
VI	16	√	√	√	16	AAAAA
	17	√	√	√	26	AAA
	18	√	√	√	26	AAA
	19	√	√	√	45	AAAAA
	20	√	√	√	55	AAA
	21	√	√	√	56	AAA
	22	√	√	√	110	AAAA
	23	√	√	√	57	AAAAA
	24	√	√	√	26	A

*The 24 antibodies were divided into five groups (I–VI) according to tissue specificity. The table also includes the molecular weight of the band from the western blot in **Figure 2** and the relative intensity of each band with the lowest defined as 1A and the highest defined as 5A.*

### Subcellular Localization of Inflorescence Proteins by Immunofluorescence

**FIGURE 3:**
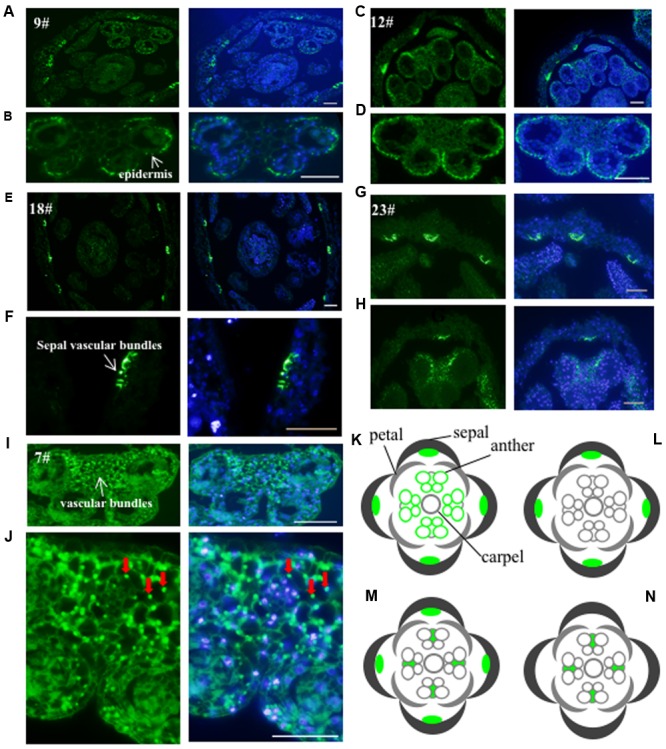
**Validation of the monoclonal antibodies by immunofluorescence.** The 24 antibodies with specific bands in the western blot were examined for immunofluorescence. Five antibodies showed specific signals and were divided into four groups. The first group including antibody No. 9 **(A,B)** and No. 12 **(C,D)** had the signals mainly in the sepal veins and epidermis of anther. A diagram indicating the positive signals was drew in **(K)**. The second group such as antibody No. 18 is only observed in sepal veins **(E,F)** and a corresponding diagram was shown in **(L)**. The signal from No. 23 **(G,H)** showed both in sepal veins and the vascular bundles of anthers with a corresponding diagram **(M)**. The signal of the last No. 7 antibody **(I,J)** was only detected in the vascular bundles of the anther with diagram shown in **(N)**. Green color indicates the positive signals marked with arrows, while the blue color shows DAPI-stained image. Bar = 10 μm.

To investigate the subcellular localization of the proteins recognized by these antibodies, we performed immunofluorescence assay on floral histological sections. The basic structure of the *Arabidopsis* flower consists of four whorls of organs from the outer to the inner: sepals, petals, stamens, and carpels (Ma, 2005). Five antibodies (No. 7, 9, 12, 18, and 23) detected cell-type dependent signals in anthers (**Figure 3**), three antibodies (No. 19, 21, and 24) showed signals in the whole floral structures (Supplementary Figure S2), while the other antibodies did not show positive signals in anthers. Based on the signal patterns recognized by the antibodies, we grouped the antigens into four categories (**Figures 3K–N**). The first group consists of antigens that were detected by No. 9 and No. 12 and localized in the sepal veins and anther epidermis (**Figures 3A–D,K**). In the second group, No.18 recognized a specific signal in the sepal veins (**Figures 3E,F,L**). In the third group, No.23 displayed signals in both the sepal veins and the vascular bundles of anthers (**Figures 3G,H,M**). The last group includes No. 7, which detected antigens in all cell types within the anther, with slightly stronger signals within the vascular bundles of the anther (**Figures 3I,J,N**). In summary, five of the antibodies (No. 7, 9, 12, 18, and 23) detected specific signals and could be used as markers for immunofluorescence assay during flower or anther development.

### Identification of the Candidate Antigens of the 24 Antibodies by Mass Spectrometry

**FIGURE 4:**
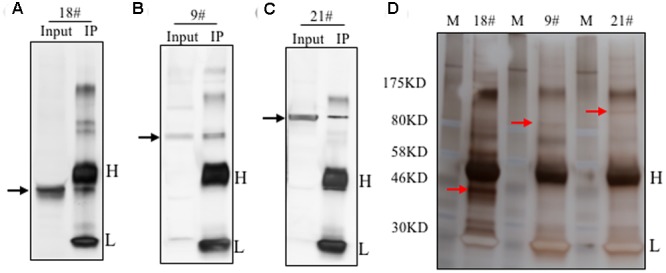
**Immunoprecipitation of the antigens using the identified antibodies.** Immunoprecipitation was performed with these 24 antibodies. The total proteins from flowers was loaded as the input. Three antibodies showed efficient performance for IP. Western blot showed here with antibody No. 18 **(A)**, No. 9 **(B)** and No. 21 **(C)**. The target band was shown by the black arrow. Silver staining result was shown in **(D)**. The targeted bands with red arrow were cut for mass spectrometry analysis.

To identify the antigens for the 24 antibodies, we conducted IP experiments with total proteins extracted from *Arabidopsis* inflorescences. The antibody-antigen complex was precipitated by protein A/G beads and then detected by WB with the same antibody used in IP. Subsequently, No. 9, 18, and 21 antibodies detected specific protein bands, whose sizes were consistent with that in input samples (**Figures 4A–C**), suggesting that the antigens of the three antibodies could be enriched by IP. Prior to analysis of the IP-enriched samples by MS (mass spectrometry), the IP-enriched samples were run on a SDS-page gel followed by silver staining (**Figure 4D**). Based on the molecular weight detected by WB, we excised the corresponding band (**Figure 4D**) for subsequent MS analysis. The candidates subsequently detected by MS are shown in **Supplementary Table S1**. The molecular weight of the antigens detected by WB and the peptide sequence revealed by MS allowed us to conclude that the corresponding antigen recognized by No. 9 was likely AT5G53170 (**Table 2**), which is an FtsH protease 11 (Sakamoto et al., 2003). The No. 18 antigen was probably AT1G11860, the glycine cleavage T-protein, which has aminomethyltransferase activity and is involved in the mitochondrial conversion of glycine to serine during the major photorespiratory pathway (Douce and Neuburger, 1999). Finally, the No. 21 antigen is most likely AT2G25140, a casein lytic proteinase B4 (Lee et al., 2007). The results for the other 7 antibodies analyzed by MS and the potential antigens for each corresponding antibody are listed in **Supplementary Table S1**. Using this approach, we identified 10 candidate antigens that can be recognized by their corresponding monoclonal antibodies. Particularly, the antigens for No. 9, 18, and 21 can be efficiently obtained using immunoprecipitation and can serve as positive markers in such experiments.

**Table 2:** Potential antigens of the three antibodies.

Antibody number	Accession	Description	Score	Unique peptides	MW (kDa)
18	AT1G11860	Glycine cleavage T-protein family	56.18	2	44.4
9	AT5G53170	FTSH protease 11	160.65	4	88.7
21	AT2G25140	casein lytic proteinase B4	67.95	1	108.6

*The detailed information from the mass spectrometry results are shown in **Supplementary Table S1**. Here we listed the possible targets for each antibody according to the molecular weight size based on western blot data*

### Functional Implication of the Three Potential Candidate Antigens

To further investigate the potential relevance of the three identified genes in relation to flower development, we checked expression data of these three genes from AtGenExpress Visualization Tool (AVT) and generated a heat map (Supplementary Figure S3). We found that genes encoding candidate antigens of No. 9 and No. 18 antibodies showed a similar expression pattern with higher levels in vegetative tissues than that of reproductive tissues such as leaves, consistent to their similar subcellular localization by immunofluorescence assay, suggesting a potential role in vegetative tissues. Indeed, the candidate antigen of No. 9 encodes an FtsH protease 11, *Arabidopsis* genome has 12 FtsH proteases, which play important role in the repair cycle of photosystem II in thylakoid membranes (Sakamoto et al., 2003). The candidate antigen of No. 18 glycine cleavage T-protein is one of the four different proteins required for glycine decarboxylase reaction in photorespiratory pathway (Peterhansel et al., 2010). These previous findings further support the similar localization of both proteins at vegetative tissues. In contrast, the gene of candidate antigen of No. 21 antibody was highly expressed in reproductive tissues rather than at vegetative tissues such as sepals, stamen, and carpels (Supplementary Figure S3), suggesting a special role in these tissues. Consistently, previous studies showed that, in comparison to the other members, mutation of *CLPB4* (No. 21) did not cause any defects in vegetative development (Lee et al., 2007), but the reproductive development in *capb4* mutant was not tested. We also checked the expression patterns of the other potential seven targets, which showed various expression at vegetative and reproductive tissues (Supplementary Figure S4), but this needs further study for confirmation.

## Discussion

In previous studies, several methods have been used to construct antibody libraries (Iba and Kurosawa, 1997). For example, phage display technology which has become a powerful method for making an antibody library (Winter et al., 1994). For antigens discovery, they fully used available genetic information for genes encoding antigens to screen the antibody pools. With the development and sensitivity of MS technique, which has been used for identification of antigen to antibody in human systemic autoimmune diseases (Cottrell et al., 2012). Each method has its advantages and disadvantages. However, it is still a major challenge to identify unknown antigens in large-scale manner.

The flower is essential for reproduction and is a unique structure in angiosperms. Flower development has been extensively studied using molecular genetic tools. However, very few antibodies are available for recognizing proteins involved in the floral development. Here, we used a systematic procedure to generate a monoclonal antibody library from total protein extracted from *Arabidopsis* flowers and hope that the identified antibodies can be used as cell-type markers to study flower development. The procedure is quite similar to the method described previously for human proteins (Cottrell et al., 2012). By WB screening, we identified 24 antibodies that each detected one specific band in the leaf, stem, or flower. We then performed an immunofluorescence microscopy assay to determine the ability of the antibodies to recognize the subcellular localization of their corresponding antigens. Consequently, five antibodies as described above displayed tissue-specific signals in the flower section. We then performed immunoprecipitation with these antibodies and determined that three of them enriched for a specific band after running a SDS-PAGE gel. Subsequent MS analysis identified the candidate antigens for three antibodies. Although the identities of the antigens need to be confirmed by further genetic experiments, the antibodies could certainly specifically recognize cellular biomarkers useful for studying flower development. Moreover, these biomarkers could potentially help in elucidating the process of flower development in other plant species.

Unfortunately, proteins with higher expression levels in flower total protein will always have a higher chance to be recognized by the immune system and thus a higher chance to stimulate production of antibodies compared to proteins with lower expression levels. Thus we may have developed several antibodies that are specific for those ubiquitous housekeeping proteins rather than organ-specific proteins.

Here, we show that this is the first time we are successfully able to produce antibodies for plant proteins using a library screen method. Interestingly, we were able to produce antibodies that were specific for certain proteins expressed in distinct plant organs from total protein extracted from the flower organ. We were also able to find some specific antibodies that bound more ubiquitous proteins, which we hypothesize to be prevalent but important housekeeping proteins. Future studies may focus on elucidating the exact function of these unknown housekeeping proteins. Further optimization may lead to the discovery of more useful antibodies that may specifically recognize proteins important for flower development. In the future studies, we can optimize the methods we used here to find more useful antibodies to study flower development in plants. This study is potentially of value and can lead to the generation of very useful molecular tools.

## Author Contributions

QS, YW, and HM designed experiments, QS and YW collected tissues. QS conducted most experiments. QS, LZ, YW, and HM wrote the paper. All authors read and approved the final manuscript.

## Conflict of Interest Statement

The authors declare that the research was conducted in the absence of any commercial or financial relationships that could be construed as a potential conflict of interest.

## Supplementary Material

Table S1 **Immunoprecipitation results detected by mass spectrometry for 10 antibodies (4#, 6#, 7#, 8#, 9#, 12#, 14#, 18#, 21#, and 22#).** Highlighted in each sheet is the possible candidate antigen.
