# Supplementary material for: A Strategy for Screening Monoclonal Antibodies for Arabidopsis Flowers
Source: Front Plant Sci. 2017 Feb 28;8:270. doi: 10.3389/fpls.2017.00270 (PMC5330178; doi:10.3389/fpls.2017.00270)

Figure S1. Full scans of western blot data as shown in Figure 2.

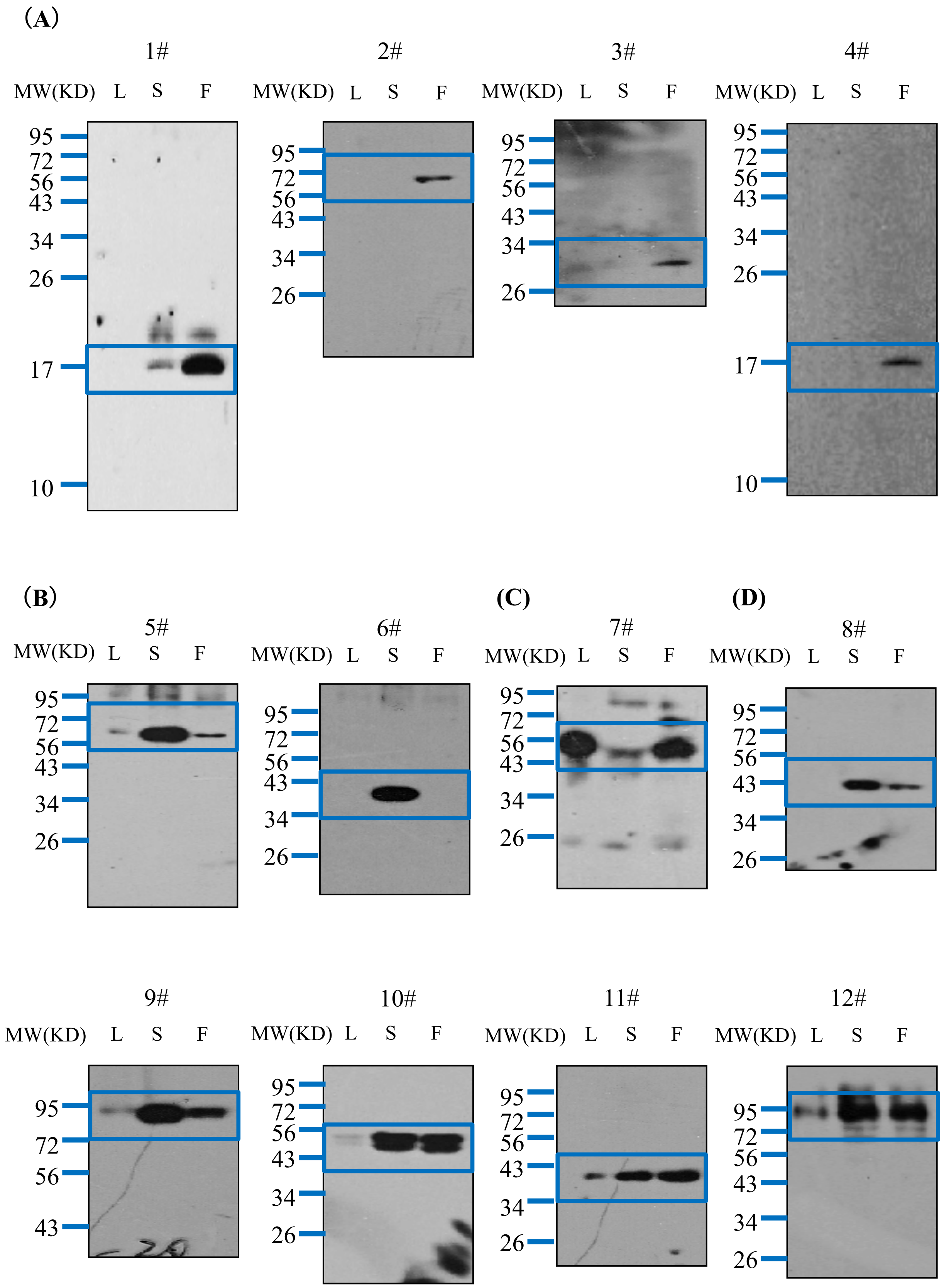

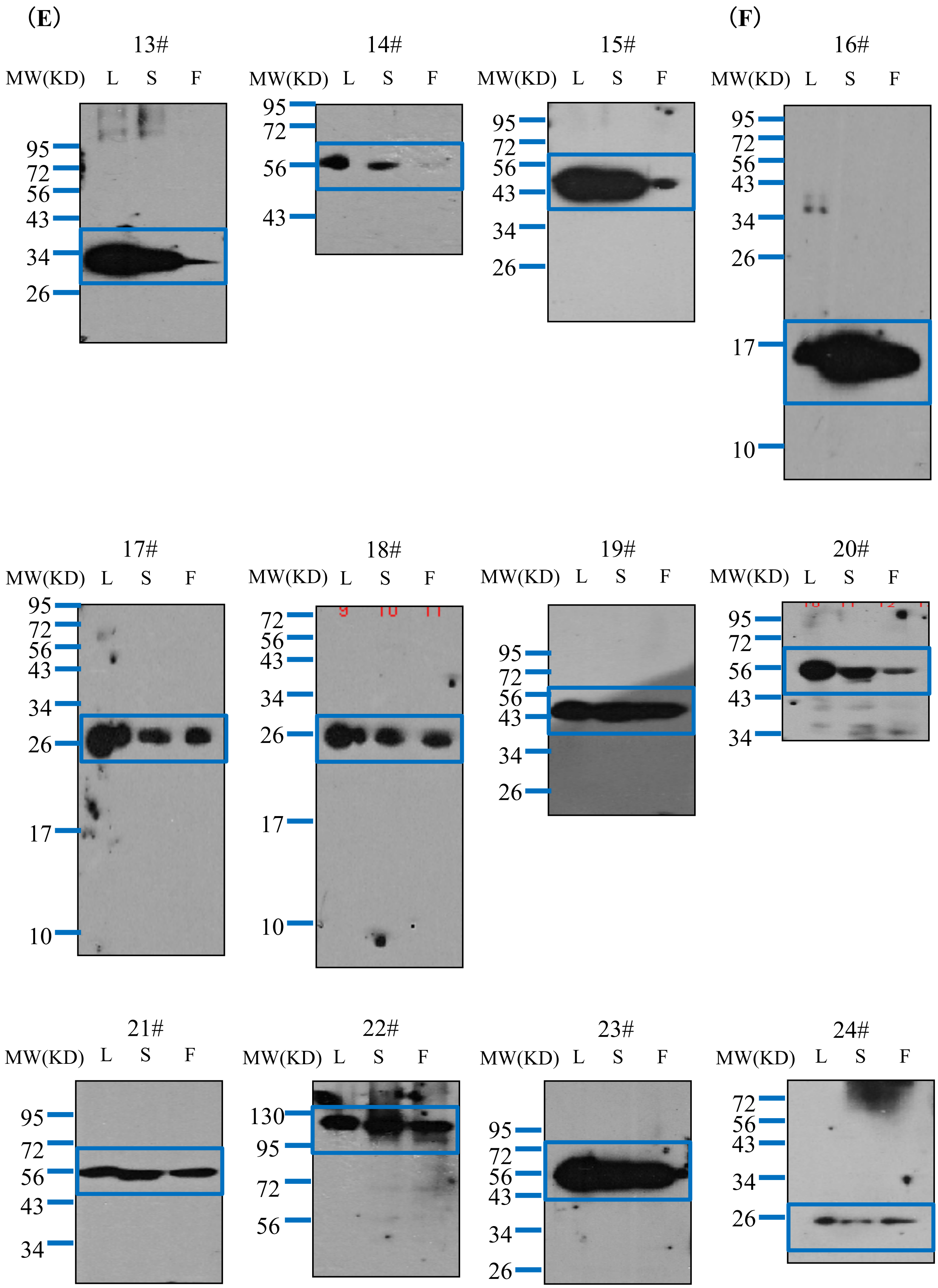
(E)
(F)
13#
14#
15#
16#
17#
18#
19#
20#
21#
22#
23#
24#
MW(KD) L S F
95
72
56
43
34
26
17
10
130

Figure S2. Immunofluorescence images of three monoclonal antibodies (19#, 21# and 24#). Bar = 10 μm.

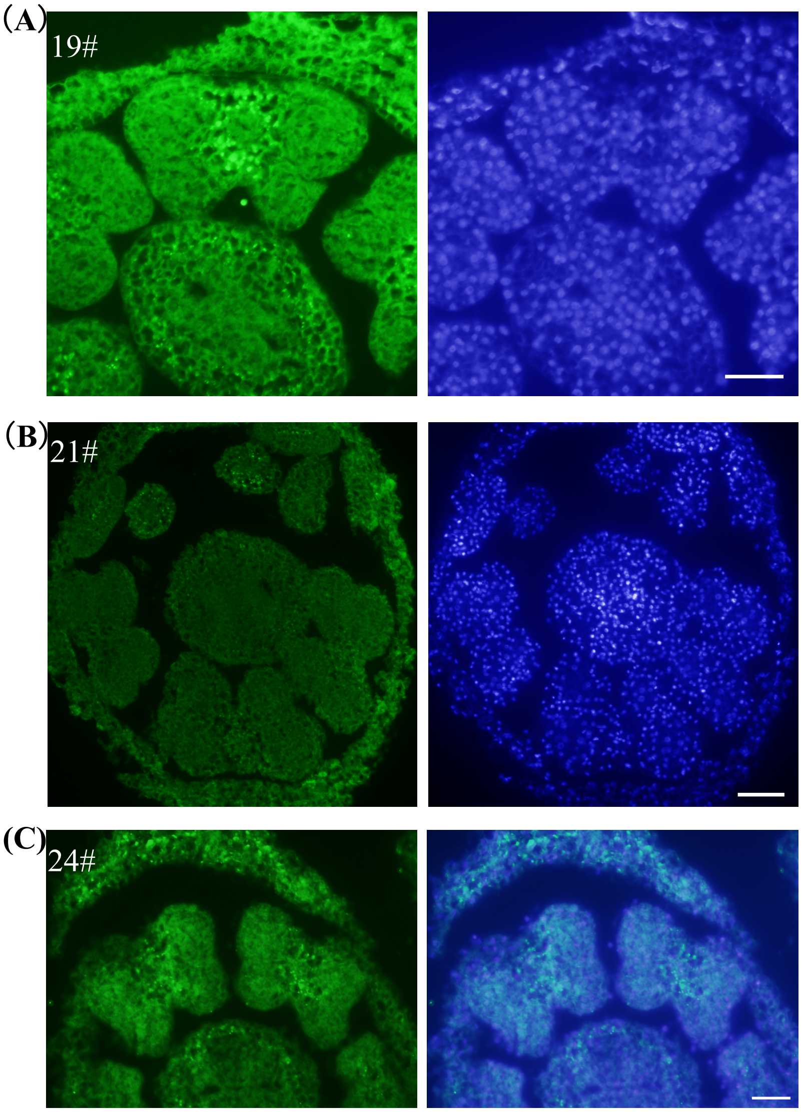

Figure S3. Heat map of expression pattern of candidate antigens for three antibodies (9#, 18# and 21#).

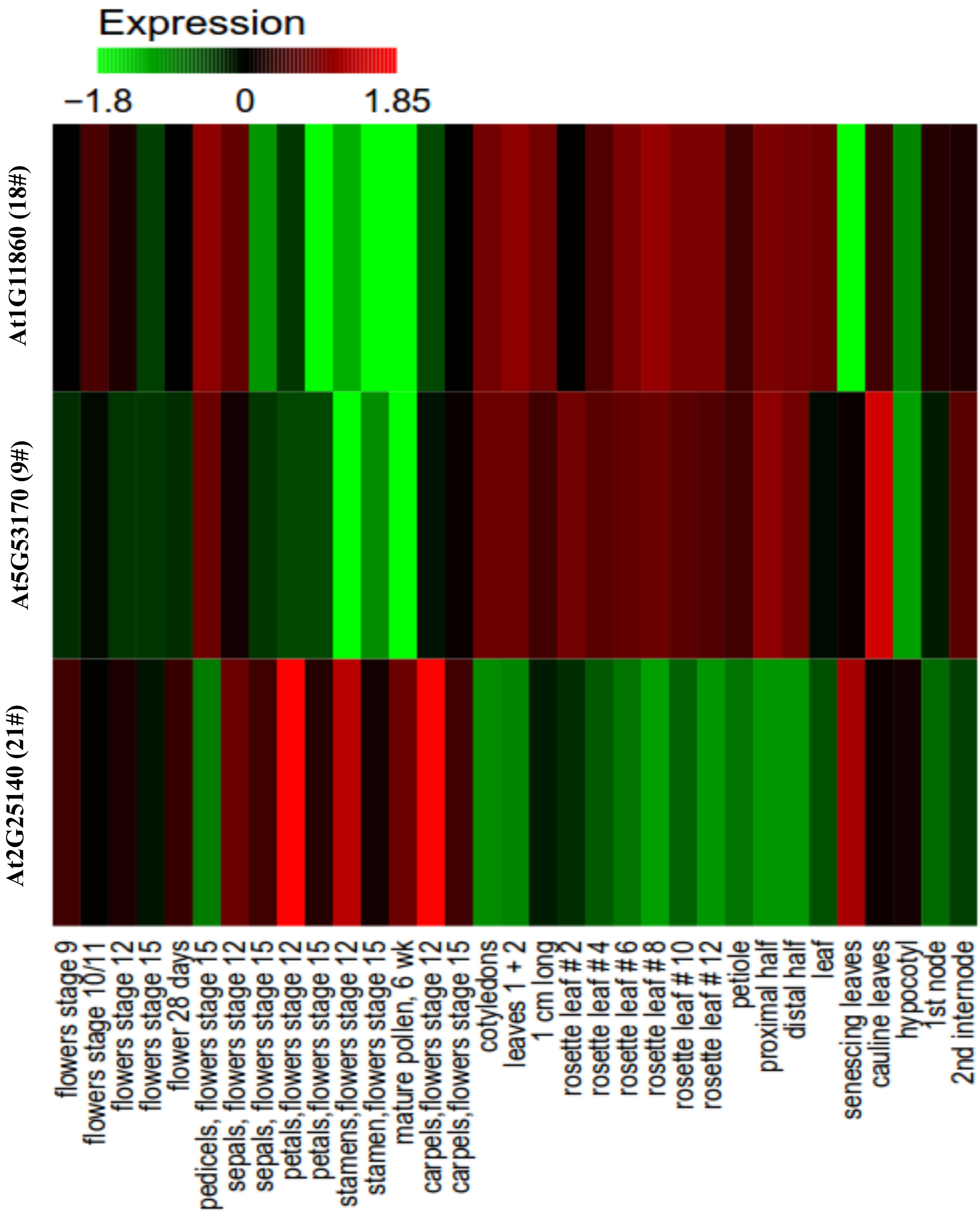

Figure S4. Heat map of the expression pattern of candidate antigens for the other seven antibodies (4#, 6#, 7#, 8#, 12#, 14# and 22#).

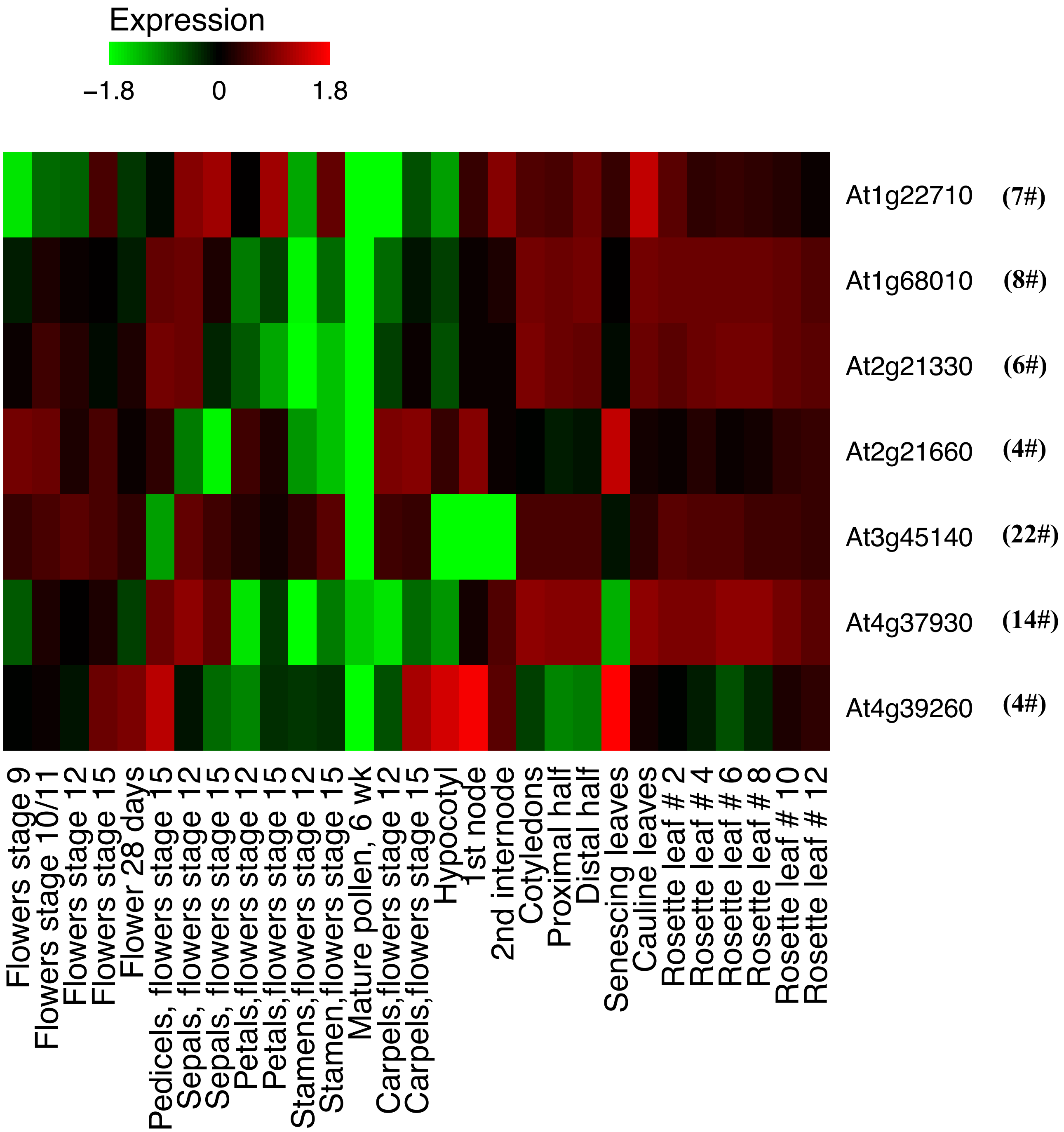

Supplement: Supplementary file 2 [file Image_1.PDF]
